# Complication rates after proximal femoral nailing: does level of training matter?

**DOI:** 10.1186/s10195-023-00737-z

**Published:** 2023-11-03

**Authors:** D. J. Haslhofer, J. M. Stiftinger, N. Kraml, F. Dannbauer, C. Schmolmüller, T. Gotterbarm, O. Kwasny, A. Klasan

**Affiliations:** 1 Department for Trauma Surgery and Sport Traumatology, Med Campus III, Kepler University Hospital Linz, Krankenhausstrasse 9, 4020 Linz, Austria; 2 Department for Orthopaedics and Traumatology, Med Campus III, Kepler University Hospital Linz, Krankenhausstrasse 9, 4020 Linz, Austria; 3 Faculty of Medicine, Johannes Kepler University Linz, Altenbergerstrasse 69, 4040 Linz, Austria; 4 Weingartshofstraße 6/609, 4020 Linz, Austria; 5 Department for Orthopedics and Traumatology, AUVA Graz, Göstinger Straße 24, 8020 Graz, Austria

**Keywords:** Complication rate, Pertrochanteric fracture, Resident training, Revision surgery, Orthogeriatrics, Proximal femoral nailing

## Abstract

**Background:**

Surgical treatment of pertrochanteric fractures is one of the most performed surgeries in orthogeriatrics. Proximal femoral nailing, the most performed procedure, is often used as a training surgery for young residents. The objective of this study was to evaluate the relevance of the resident’s training level to complication rates.

**Material and methods:**

This study was a retrospective cohort study. Surgeons were divided into four groups according to their training level. Complications included infection, cut-out, and revision surgery. The study was performed at a level 1 trauma center. All patients who were treated with proximal femoral nailing surgery with a radiological follow-up of at least 3 months were included.

**Results:**

Of the 955 patients extracted, a total of 564 patients met the inclusion criteria. Second-year residents had significantly higher cut-out rates (*p* = 0.012). Further analysis indicated a correlation between level of training and surgery duration (*p* < 0.001) as well as a correlation between surgery duration and infection rate (*p* < 0.001). The overall complication rate was 11.2%. Analyzing overall complications, no significant difference was found when comparing surgeon groups (*p* = 0.3). No statistically significant difference was found concerning infection (*p* = 0.6), cut-out (*p* = 0.7), and revision surgery (*p* = 0.3) either.

**Conclusion:**

Complication rates after proximal femoral nailing are not higher in patients who are treated by residents. Therefore, proximal femoral nailing is an excellent procedure for general orthopedic training. However, we must keep in mind that accurate positioning of the femoral neck screw is essential to keep cut-out rates as low as possible.

*Level of Evidence* III.

## Introduction

Due to ever-increasing life expectancy, the proportion of elderly people in our society is increasing. Therefore, the number of common injuries in this age group, such as hip fractures, continues to grow, and an adequate therapy regime and optimal treatment are becoming more and more important [1]. Predictions indicate that the number of hip fractures worldwide will reach 6 million per year in 2040 [2].

In Germany, approximately 74,000 fractures per year occur in the pertrochanteric area [3]. Between 2009 and 2019, the incidence increased by 24% [3].

Surgery is the only reasonable treatment when suffering such trauma. In continental Europe, the preferred surgical procedure for these fractures is proximal femoral nailing (PFN), which is significantly more commonly used than the dynamic hip screw (DHS) [4–7]. This kind of surgical procedure is performed by trauma surgeons and is a commonly used and suitable training surgery for less-experienced residents to acquire general surgical skills due to its standardized process and high incidence [8, 9].

There are few publications comparing revision rates of residents and senior surgeons. Most of them are from general surgery and show similar results. Most of those studies failed to show a significant difference in complication rates based on experience [10–13]. In orthopedics, there are no studies that have discussed the exact level of training of residents (in years to senior surgeon status) in relation to revision rates.

Patient safety issues always lead to the question of whether complication rates are higher when surgeries are performed by residents. Therefore, we took a closer look at the different training levels of residents and their impact on complications and other parameters.

The aim of this study was to evaluate the relevance of the level of the resident’s training to the outcome and revision rate in patients with proximal femoral nailing of pertrochanteric fractures. It was hypothesized that less experience would result in higher complication rates.

## Patients and methods

### Ethics

The study was approved by the local regional ethical committee (ECS 1191/2021).

### Patients

Consecutive patients with an isolated pertrochanteric fracture treated with a proximal femoral nailing surgery in our level-1 trauma center between 1 January 2016 and 30 April 2021 were included. We excluded patients without at least a 3-month postoperative radiograph and patients with concomitant fractures that required further surgeries.

**Fig. 1:**
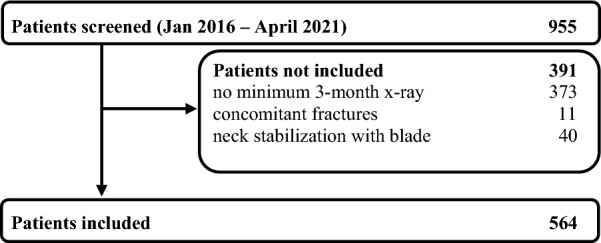
Patient's flowchart

The data for 955 consecutive patients were extracted from this period (Fig. 1).

We collected age, gender, side of injury, body mass index (BMI), and fracture classification according to the AO classification (31-A1 to 31-A3) [14]. We further collected intraoperative data—surgery date, surgeon, surgery duration, surgery start time, nail type and nail diameter, femoral neck implant, femoral neck implant length, and intraoperative radiological position of the femoral neck implant.

Postoperative complications were defined as infection (positive postoperative wound swab) or cut-out and revision surgery for any reason.

Radiographic evaluation was performed based on preoperative X-ray images. Furthermore, the position of the neck screw as well as signs of a cut-out were examined in the most recent images for each patient.

### Methods

To investigate the effects of level of training, the surgeons were assigned to four groups. Depending on the year of training of the residents at the time of surgery, they were assigned to groups 1–3 (group 1—training year 1 + 2, group 2—training year 3 + 4, group 3—training year 5 + 6). Senior surgeons were assigned to group 4.

Residency typically lasts 6 years. Before entering the training period, 9 months of basic training must be completed, at least 3 months of which must be in a surgical specialty. During this phase, however, the trainee is not yet involved in active surgical activities. For surgeons with prior surgical experience, the level of training is adjusted accordingly.

Surgery was performed with the patient in the supine position on an extension table. Preoperative single-shot antibiotics with 1.5 g cephazolin were standardized. After a reduction under fluoroscopy on both projections, the surgical area was prepped and sterilely draped. The surgical procedure corresponded to the procedures specified for the particular type of nail used. Open reduction was only performed when an anatomical reduction via closed reduction could not be achieved. Cerclage cables were used when the type of fracture needed extra stabilization. Postoperatively, thromboembolic prophylaxis with a low molecular weight heparin was administered for at least 10 days. Postoperative antibiotics were not used as standard. Full weight-bearing was possible immediately if the surgical treatment allowed it.

A senior surgeon was always present in the operating room during the first operations performed by residents. The transition, where the residents finally perform the operation without direct supervision, is completed before the end of the second-year training. After this transition, the senior surgeon is on standby, only getting involved if the trainee calls for the surgeon. This has the significant teaching benefit of increasing the responsibility held by the trainee. Fluoroscopic images showing the k-wire positioned inside the femoral neck were always supervised by senior surgeons during this transition time. Residents in advanced training years discussed the postoperative X-rays with a senior.

**Fig. 2:**
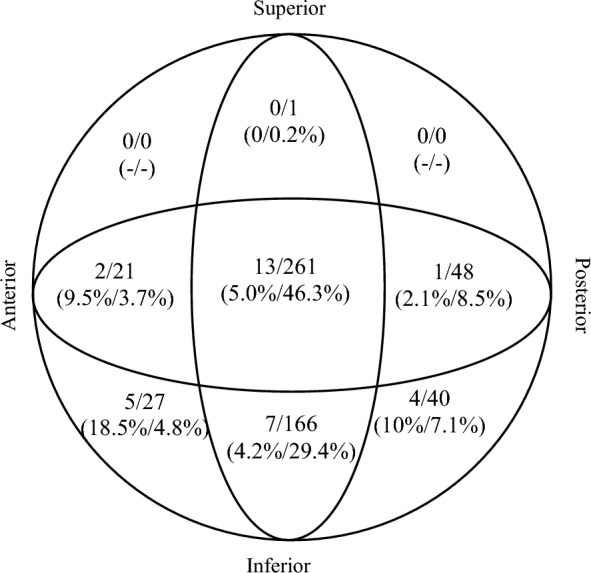
Screw positioning within the femoral neck. The first number describes the cut-out cases, and the second number describes the total amount of screws in the particular area. The first percentage is the cut-out rate in each area, and the second percentage is the overall position rate

The assessment of the position of the femoral neck screw in the intraoperative fluoroscopic images was performed in anterior–posterior view as well as in axial view. For this purpose, the femoral neck was divided into thirds in each view, and thus the exact position was determined. Consequently, there were nine possible positions of the femoral neck screw (Fig. 2). Calculation of the tip apex distance (TAD) was also performed using intraoperative fluoroscopic images.

Classification of the fracture type was performed by four of the authors, two seniors, and two residents using the AO classification (31-A1 to 31-A3). Both inter- and intraobserver ICCs were high, 0.92 and 0.96.

The following incidents were included as complications: infections, defined as at least one documented positive wound swab; cut-out, identified in correlating fluorographs or computer tomography scans; and revision surgery, defined as documented unplanned surgery subsequent to the initial surgery provided [15].

### Statistics

Statistical analysis was performed using IBM SPSS Statistics 28 (Chicago, IL, USA). Data are reported as mean (± SD) for normally distributed data and median [IQR] for non-normally distributed data. To test our hypothesis, we used the Pearson’s chi-square test. Significance was set at *p* < 0.05.

## Results

Of the 955 extracted patients, a total of 564 patients met the inclusion criteria—398 females (70.6%) and 166 males (29.4%). Age reached from 35 to 98 years, with a mean of 80.7 years. Mean body mass index (BMI) was 24.3 (minimum 13, maximum 40); see Table 1.

**Table 1:** Demographic data

	Total	Group 1 (residents in their 1st or 2nd year)	Group 2 (residents in their 3rd or 4th year)	Group 3 (residents in their 5th or 6th year)	Group 4 (senior surgeons)
Total	564 (100%)	106 (18.8%)	91 (16.1%)	94 (16.7%)	273 (48.4%)
Gender					
Female	398 (70.6%)	71 (67.0%)	74 (81.3%)	71 (75.5%)	182 (66.7%)
Male	166 (29.4%)	35 (33.0%)	17 (18.7%)	23 (24.5%)	91 (33.3%)
Age					
Years	80.7 (35–98)	82.3 (50–96)	80.3 (50–96)	80.8 (57–96)	80.1 (35–98)
BMI					
kg/m^2^	24.3 (13–40)	23.9 (16–40)	24.1 (16–35)	24.2 (13–36)	24.6 (14–39)
Side					
Right	279 (49.5%)	52 (49.1%)	51 (56.0%)	41 (43.6%)	135 (49.5%)
Left	285 (50.5%)	54 (50.9%)	40 (44.0%)	53 (56.4%)	138 (50.5%)
AO classification					
31-A1	129 (22.9%)	33 (31.1%)	17 (18.7%)	16 (17.0%)	63 (23.1%)
31-A2	352 (62.4%)	64 (60.4%)	64 (70.3%)	64 (68.1%)	160 (58.6%)
31-A3	83 (14.7%)	9 (8.5%)	10 (11.0%)	14 (14.9%)	50 (18.3%)
Surgery duration					
Minutes	55.6 (23–173)	65.4 (31–173)	62.2 (35–142)	54.1 (23–128)	50.2 (24–165)
Type of nail					
TFN	294 (52.1%)	38 (35.8%)	37 (40.7%)	58 (61.7%)	161 (59.0%)
TFN-A	110 (19.5%)	34 (32.1%)	30 (33.0%)	9 (9.6%)	37 (13.6%)
Gamma	160 (28.4%)	34 (32.1%)	24 (26.4%)	27 (28.7%)	75 (27.5%)

Fractures of AO classification 31-A2 had the highest incidence (62.4%). Supplementary care using cerclage cable was necessary in 33 patients (5.9%). Senior surgeons performed 48.4% of the surgeries. The remaining surgeries were almost evenly distributed into the other groups (16.1–18.8%). Mean surgery duration was 55.6 min (IQR = 25).

Screw positioning was center-center in the femoral neck in 261 patients (46.3%). An inferior-center position occurred in 29.4% (166 patients). The other 137 screws were positioned in other areas (24.3%); see Fig. 2. TAD was measured to be greater than 25 mm in 64 patients (11.3%). Cut-out rates were significantly higher in patients with a TAD > 25 mm (*p* = 0.002). No correlation between level of training and higher TAD was indicated (*p* = 0.5).

The data showed that second-year residents had significantly higher cut-out rates (*p* = 0.012). A correlation between level of training and surgery duration (*p* < 0.001) was indicated, as was a correlation between surgery duration and infection rate (*p* < 0.001).

In total, complications occurred in 63 patients (11.2%). Postoperative infections were detected in 20 cases (3.5%). Thirty-two cut-outs developed (5.7%). Revision surgery was necessary in 56 patients (9.9%); see Table 2.

**Table 2:** Complications

	*n*	%
Complications	63	11.2
Postoperative infection	20	3.5
Cut-out	32	5.7
Revision surgery	56	9.9

Most of the revision surgeries were a change to a hemiarthroplasty (26.8%). Other revision surgeries were debridement or wound revision, removal/replacement of the femoral neck screw, replacement/reattachment of the nail, conversion to a total hip arthroplasty, or others (Table 3). Revision surgery occurred a mean of 102 days after the initial intervention (IQR = 43).

**Table 3:** Revision surgeries

	*n*	% of the revision group	% overall
Change to hemiarthroplasty	15	26.8	2.7
Debridement and wound revision	9	16.1	1.6
Removal/replacement of femoral neck screw	8	14.3	1.4
Periosteosynthetic fracture	5	8.9	0.9
Replacement/reattachment of the nail	9	16.1	1.6
Change to total hip arthroplasty (THA)	4	7.1	0.7
Partial metal removal	1	1.8	0.2
Implant removal	3	5.4	0.5
Dynamization	3	5.4	0.5

The hypothesis was refuted since no statistically significant difference could be shown that correlated the level of training to complications (*p* = 0.3). The same results occurred concerning revision surgery (*p* = 0.3) and postoperative infections (*p* = 0.6). When the correlation between level of training and cut-outs was examined, no statistical significance was found (*p* = 0.7); see Table 4.

**Table 4:** Relationship of complications to level of training

	Significance (test method)
Complications	*p* = 0.3 (ns) (*χ*^2^ test)
Revision surgery	*p* = 0.3 (ns) (*χ*^2^ test)
Postoperative infections	*p* = 0.6 (ns) (*χ*^2^ test)
Cut-outs	*p* = 0.7 (ns) (*χ*^2^ test)

*ns* not significant; *χ*^*2*^* test* Pearson’s chi-square test

Further classification (into groups 1–6 for residents, depending on their year of training, and group 7 for senior surgeons) showed no statistically significant difference between groups (*p* = 0.5).

No statistically significant difference was seen concerning the distribution of different fracture types between the training groups (*p* = 0.2).

The use of cerclage cables was associated with a higher overall complication rate (*p* = 0.014) but not with a higher risk of cut-out (*p* = 0.4).

Data also showed no correlation between time of surgery and complications (*p* = 0.6). A higher BMI was associated with higher infection rates (*p* = 0.006).

## Discussion

This study could not show any statistically significant difference concerning the correlation of the level of training with complication rates. Analyzing complications in detail, we demonstrated that our group of second-year residents had significantly higher cut-out rates. Possible reasons could be excessive resident confidence during this period of residency (leading to lower concentration and greater inaccuracy) and the possible change to partly independent surgeries.

The optimal position of the femoral neck screw is one of the most important factors in the mechanical stability of the osteosynthesis. In various studies, a center-center or inferior-center position is recommended [16, 17]. On the other hand, superior and/or anterior positioning are described as critical and increase the risk of cut-out. The data from our study are in line with these statements, as they also show increased cut-out rates in cases in which screws were malpositioned. With a cut-out rate of anteriorly positioned screws of about 10–20%, we show similar results to Jiamton et al. [18]. The TAD is deemed to be another important factor in stability. Baumgaertner et al. stated that a TAD of 25 mm is the cutoff for a higher risk of cut-out [19]. Our study shows results which support this theory.

Complication rates have been published in various studies of intramedullary femoral nailing. Overall complication rates in other studies are similar to ours (7.9–14.9%) [8, 9, 20].

In more detail, infection rates reach from 2.7 to 9.6% in various publications and a meta-analysis [8, 20–23]. Our data showed infection rates of 3.5%. Concerning revision surgery, we show similar results to other studies (9.9% vs. 4.3–14%) [20, 22, 24]. Our findings demonstrated comparable cut-out rates as well (5.7% vs. 1.2–6.9%) [8, 20, 22].

Our results support the data about proximal femoral nailing as a teaching surgery published by Biber et al. and Schütze et al. [9, 25]. Like our study, they did not show a significant link between procedures performed by residents and higher complication rates.

Our study expanded on this knowledge by investigating, in detail, the level of training (in years) and its impact on surgical outcome.

Comparing our data on training-dependent complication rates with studies from other medical specialties, in particular abdominal surgery, and general surgery, we find higher complication rates for the various procedures in some cases. However, as in our study, a comparison between surgeries performed by residents and those performed by senior physicians did not show any statistically significant differences in complication rates [11–13].

Nowadays, when the teaching of a surgical specialty is discussed, off-patient training must not be left out. Cadaveric simulations of various procedures lead to the enhancement of both technical and nontechnical skills [26, 27]. Surgical simulators are another tool for improving different surgical skills. Froelich et al. showed that the positioning of a single central guide pin into a femoral neck improved and less fluoroscopic time was needed to perform this procedure after using a surgical simulator with haptic feedback [28]. These are just two possibilities for off-patient teaching of young residents to improve their skills.

A weakness of our retrospectively conducted study is that only about 60% (582 patients) of the injured patients who were treated had at least one follow-up examination performed after 3 months. This is mostly due to the advanced age of the patients. Mortality is high in this cohort, and follow-ups are difficult to perform due to the general frailty of the patients.

The training concept used for this specific procedure in this institution is only one possible approach, so the results are not generalizable. In various centers and countries, due to legal-ethical considerations, a senior surgeon always has to be present throughout the procedure, which might alter the results significantly.

## Conclusion

Complication rates after proximal femoral nailing are not higher in patients who are treated by residents. Therefore, proximal femoral nailing is an excellent procedure for general orthopedic training. However, we must keep in mind that accurate positioning of the femoral neck screw is essential to keep cut-out rates as low as possible.

## Data Availability

Not applicable.
